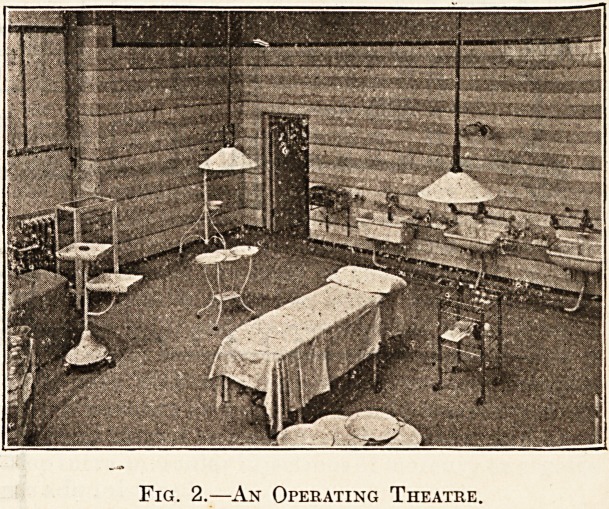# The Units of General Hospital Construction

**Published:** 1907-05-25

**Authors:** 


### HOSPITAL ADMINISTRATION.

### CONSTRUCTION AND ECONOMICS.

### THE UNITS OF GENERAL HOSPITAL CONSTRUCTION.

#### THE SURGICAL WARD UNIT.

The scheme of ward units is quite a departure from the old system of hospital administration, and is, undoubtedly, more expensive in construction. This is exemplified particularly in connection with the surgical unit. Previously, one, or at most two, operating theatres were considered sufficient for any hospital, and the members of the surgical staff had the use of the theatre in turn. Although, on the ground of economy in construction much can be said in favour of this arrangement, it has obvious disadvantages. Patients are frequently kept waiting for operation when the theatre is not available; and where the clinical instruction of large numbers of students is involved, the lack of accommodation seriously hampers efficient teaching. But the main disadvantage arises from the fact that in the desire to accommodate these students, the buildings are so constructed and arranged that it is quite impossible to ensure thorough cleanliness. Moreover, the developments of aseptic surgery and the increased number of operations now performed, render the provision of additional theatres an absolute necessity. Hence the justification for including an operating theatre in each surgical ward unit. The surgical differs from the medical unit, then, in the addition of an operating theatre, with anæsthetic-room, rooms for sterilising surgical dressings and storing splints, and, where possible, a small male ward of from 12 to 16 beds for the treatment of accidents. By the provision of this accident ward patients in the general ward who may have undergone serious operation are not liable to be disturbed by the admission of accident cases, at any hour of night or day. In a hospital which is not a teaching school this additional ward is not essential; it might there be found more convenient and more economical to set aside a separate unit of surgical wards for the treatment of accident cases. The accident ward, although smaller than the general male ward, does not differ from it in any essential detail. It should have the same sanitary annexes, bathroom and lavatory accommodation, ward kitchen, scullery, linen-room and day-room for convalescents. The sister's room, washing and examination room, test-room, visiting surgeon's room, and resident's quarters, are common to the unit.

The Surgical Ward Unit on Scotch System.

The surgical ward unit should be so placed that the operating theatre will have a north aspect and roof light as well as windows. The accompanying plan shows such a surgical ward unit.

The main wards, as in the medical unit, run north and south, the small ward for accidents is to the west, and on the east is a corridor communicating with the other parts of the hospital. Leading from this corridor on the north is the operating-theatre with its various rooms, and on the south a lecture room is shown which may be used either in connection with the surgical unit on the one side, or the medical unit on the other.

Within recent years, both in this country and abroad large sums of money have been spent on the erection of operating theatres. Every up-to-date hospital, irrespective of its size, has an operating-theatre fully equipped with all the latest appliances, although it may not be in use for days, or even weeks at a time, and the capital sum expended may be out of proportion to the requirements. In some hospitals theatres have been built so elaborate that they defeat what should be the main object, namely, the possibility of having thorough cleanliness in every corner. In designing an operating theatre the first point to determine is whether or not provision is to be made for students, and if so, what number. No matter how the space for students be arranged, only a limited number can have a satisfactory view of an operation. In designing an aseptic theatre, therefore, the accommodation for students should be limited to 50 as a maximum. In the interest of patients as well as students, no class should exceed this number. The old type of operating theatre of thirty years ago provided for two or three hundred students, had a wooden floor and fixed benches, and no doubt served many useful purposes. But for modern surgical work it is quite impossible. The modern operating theatre is much reduced in size, and is so constructed that it can be thoroughly cleansed from floor to ceiling.

The provision for the accommodation of students in an operating theatre is a most difficult problem to solve. In the attempt to keep the area of the theatre aseptic, where the patient, the surgeon, and his assistants and nurses are at work, the student has in many cases been put in such a position that it is with difficulty he can see any detail. Mirrors placed at an angle over the operating table have been tried, in order that students who occupy back benches might obtain a better view. It is difficult enough for a student to follow the details of an operation without having to calculate that what he sees in the mirror as left is really right, apart from the danger of hanging so weighty a body from the ceiling over the patient; as a result this arrangement has found but little favour. If we adopt the view that provision should only be made for 50 students, then how may that number be most advantageously arranged for? The plan and section show two rows of seats under the vertical light, and a gallery on each side, also with two rows. These latter are sufficiently high to permit of an uninterrupted view, and the students under the window have also the advantage of a direct view, without obstructing the light. Each student has a lifting seat of simple construction.

In the attempt to secure a really aseptic theatre nearly every variety of material has been experimented with. In some continental hospitals large glass slabs have been used in the construction of both floors and walls, but experience has proved that glass is quite unsuitable. When the slabs are placed close enough to prevent dust collecting between the joints, the glass cracks with variations of temperature. Glass tiles are open to the same objection. Marble slabs have been adopted both at home and abroad; but probably the best material for walls is a highly glazed fireclay tile, for the roof a hard cement or adamant plaster, well polished and coated with enamel paint, and for the floors marble terrazzo well laid on concrete, the concrete being allowed to solidify before the terrazzo is put on. If the terrazzo be laid before the concrete has had time to thoroughly set, and be exposed to changes of temperature, cracks are apt to occur, which may not be visible for some time. The floor of the area should have a slight incline and slope towards a gutter formed in the terrazzo, into which all the basins discharge and the waste water is drained away. This gutter is fitted with a special trap which can be easily inspected and cleaned. Fig. 2 shows the area of such an operating theatre. The walls are tiled, and there are no pipes or projections to be seen. The pipes are all exposed on the wall of the sterilising room behind the theatre, and come straight through the wall to the various fittings, thus reducing the amount of surface where dust might collect. The artificial lighting requires careful consideration.

Fig. 2.—An Operating Theatre.

No light should be fixed immediately over the operating table. Two groups of incandescent lamps with large reflectors should be placed on each side of the table, as shown in fig. 2. Each of these fittings should be provided with a separate fuse, in order to avoid the possibility of both lights going out at the same time. They should be arranged to raise and lower, and the reflectors should be constructed so that they can be fixed at any angle.

*(To be continued.)*

## Figures and Tables

**Figure f1:**
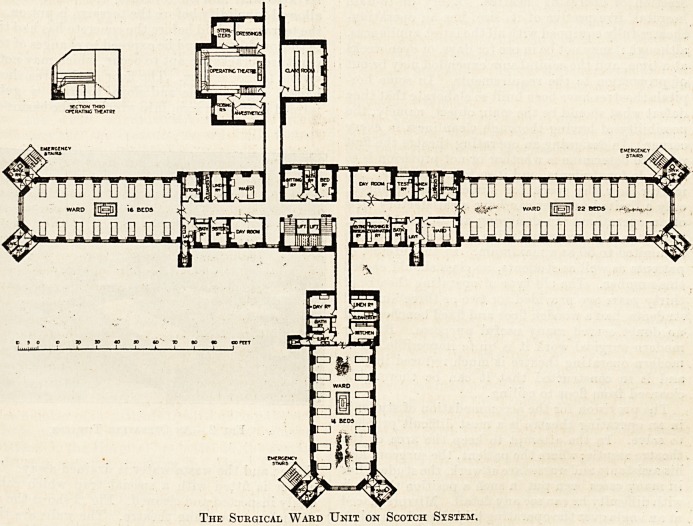


**Fig. 2. f2:**